# Improving the pharmacokinetics, biodistribution and plasma stability of monobodies

**DOI:** 10.3389/fphar.2024.1393112

**Published:** 2024-04-04

**Authors:** Adrian Valentin Dinh-Fricke, Oliver Hantschel

**Affiliations:** Institute of Physiological Chemistry, Philipps-University of Marburg, Marburg, Germany

**Keywords:** monobody, targeted therapies, pharmacokinetics, albumin binding domain, protein engineering, protein therapeutics

## Abstract

Cancer is a leading cause of death worldwide. Several targeted anticancer drugs entered clinical practice and improved survival of cancer patients with selected tumor types, but therapy resistance and metastatic disease remains a challenge. A major class of targeted anticancer drugs are therapeutic antibodies, but their use is limited to extracellular targets. Hence, alternative binding scaffolds have been investigated for intracellular use and better tumor tissue penetration. Among those, monobodies are small synthetic protein binders that were engineered to bind with high affinity and selectivity to central intracellular oncoproteins and inhibit their signaling. Despite their use as basic research tools, the potential of monobodies as protein therapeutics remains to be explored. In particular, the pharmacological properties of monobodies, including plasma stability, toxicity and pharmacokinetics have not been investigated. Here, we show that monobodies have high plasma stability, are well-tolerated in mice, but have a short half-life *in vivo* due to rapid renal clearance. Therefore, we engineered monobody fusions with an albumin-binding domain (ABD), which showed enhanced pharmacological properties without affecting their target binding: We found that ABD-monobody fusions display increased stability in mouse plasma. Most importantly, ABD-monobodies have a dramatically prolonged *in vivo* half-life and are not rapidly excreted by renal clearance, remaining in the blood significantly longer, while not accumulating in specific internal organs. Our results demonstrate the promise and versatility of monobodies to be developed into future therapeutics for cancer treatment. We anticipate that monobodies may be able to extend the spectrum of intracellular targets, resulting in a significant benefit to patient outcome.

## 1 Introduction

Cancer development is driven by the activation of oncogenes and/or inactivation of tumor suppressor genes, resulting in uncontrolled cell proliferation, prevention of cell death and evasion from immune destruction (Futreal et al., 2004; Hanahan and Weinberg, 2011). Traditional cancer treatment by surgery followed by radiation and/or chemotherapy often is not able to cure patients, due to the emergence of metastatic disease (Lambert et al., 2017; Gerstberger et al., 2023). Overall survival of cancer patients is still low for major tumor types, also due to therapy-related toxicities (Gerstberger et al., 2023). Therefore, over the past 20 years, several targeted anticancer drugs entered clinical practice, which act specifically on major oncogenic drivers in solid tumors and hematological malignancies. These drugs come in two major classes: Firstly, therapeutic antibodies can block cell surface receptors or their ligands. Secondly, small-molecule chemical inhibitors can inhibit signaling molecules, mainly protein kinases, that are critical for cell signaling inside tumor cells (Min and Lee, 2022; Roskoski, 2023). While some targeted cancer therapeutics led to therapeutic breakthroughs in specific cancer types, many of these drugs suffer from short-lived responses due to adaptive and evasive drug resistance (Konieczkowski et al., 2018; Osman and Deininger, 2021). In addition, a large number of oncoproteins remain untargeted due to the lack of deep binding pockets, which can be targeted readily with small-molecule inhibitors (Bushweller, 2019; Moore et al., 2020).

Various small engineered binding proteins, which are based on stable non-immunoglobulin scaffolds, were developed over the past 20 years (Vazquez-Lombardi et al., 2015). Besides their utility and broad application as research tools for structural, cell and molecular biology, intensive efforts to develop them as next-generation protein therapeutics for cancer and other diseases are ongoing (Hantschel, 2017; Gebauer and Skerra, 2020; Hantschel et al., 2020). Some of these protein binder classes, such as DARPins, affibodies and anticalins, reached clinical development stage. Their small size (∼8–20 kDa), rapid generation by directed evolution techniques, high affinity target binding and facile recombinant production offers great therapeutic opportunities (Luo et al., 2022). Among the most commonly used small non-immunoglobulin scaffolds are monobodies (Mb), which are generated from large combinatorial libraries using the tenth fibronectin III domain (FN3) of human fibronectin as molecular scaffold of only 94 amino acid and a molecular weight of ∼10 kDa (Koide et al., 1998; Koide et al., 2012). Since their first report, analogs of the initial monobody, including adnectins, tenascins and centyrins were developed in the pharma and biotech industries for several extracellular targets, e.g., VEGFR2, EGFR, PCSK9 (Sha et al., 2017; Gebauer and Skerra, 2020; Hantschel et al., 2020). But no monobody-drug reached clinical approval to date due to lack of efficacy and/or dose-limiting toxicities in phase I-II clinical trials (Schiff et al., 2015).

We and others have developed monobodies targeting central intracellular oncogenes, including Bcr-Abl and Src kinases, SHP-2 tyrosine phosphatase, STAT3 transcription factor, WDR5 chromatin reader and H-/K-Ras small GTPases (Wojcik et al., 2010; Grebien et al., 2011; Sha et al., 2013; Wojcik et al., 2016; Kukenshoner et al., 2017; Spencer-Smith et al., 2017; Gupta et al., 2018; La Sala et al., 2020). Upon genetic expression of monobodies in tumor cells, selective inhibition of oncoprotein-dependent signaling was observed. To enable therapeutic translation, we are developing technologies for intracellular monobody protein delivery (Hantschel, 2017; Schmit et al., 2019). Sufficient plasma stability and pharmacokinetics is a crucial prerequisite to enable efficient intracellular delivery and targeting of tumor cells *in vivo*. Therefore, we needed to investigate, and possibly improve, the stability and pharmacokinetics of monobodies and assess compatibility with parallel efforts to enable cellular delivery of monobodies.

A short plasma half-life and rapid renal clearance are common features of small biotherapeutics, which would require frequent dosing to ensure a sufficiently high drug exposure of the targeted cells/tissues and represent a significant hurdle in drug development (Gebauer and Skerra, 2020). Consequently, a number of strategies have been developed to prolong half-life of pharmaceutically-active peptides and proteins (Kontermann, 2016; Hober et al., 2019). Chemical conjugation with synthetic poly-ethylene glycol (PEG) polymers (PEGylation) has been commonly used to achieve half-life extension of clinically approved protein and peptide drugs (Gao et al., 2024). PEGylation decreases glomerular filtration by increasing the hydrodynamic radius of the biotherapeutic. On the other hand, it may result in loss of biological activity as PEG polymers are covalently attached to e.g., all lysine residues or to specific engineered cysteine positions, and may thereby sterically hinder target binding. Alternatively, genetically encodable intrinsically unstructured amino acid sequences, with similar properties to PEG, can be fused to biopharmaceuticals to increase their hydrodynamic radius. Among the most commonly used genetically encodable sequences are the XTEN and PASylation sequences (Schellenberger et al., 2009; Schlapschy et al., 2013). But fusion of these unstructured sequences may negatively impact monobody expression and ease of purification. PEGylation was previously used to improve pharmacological properties of the VEGFR2-targeting adnectin CT-322, but due to lack of efficacy in recurrent glioblastoma in a phase II clinical trial, development was stopped (Mamluk et al., 2010; Schiff et al., 2015). In addition, a preclinical study showed that PASylation of CT-322 was only able to moderately increase half-life in mice by ∼4-fold (Aghaabdollahian et al., 2019). These strategies, which increase the size of monobodies by several-fold, are hard to envisage to be compatible with intracellular delivery strategies, where smaller size of the cargo protein often resulted in more efficient cellular uptake.

Another strategy to increase half-life and improve other pharmacokinetic parameters of small protein binders is centered around albumin (Kontermann, 2016). Direct fusion to albumin has successfully been employed to improve the pharmacokinetics of a bispecific CEA + CD3 antibody and is a common strategy to increase the half-life of recombinant cytokines and coagulation factors (Muller et al., 2007; Schulte, 2013). Apart from the increased size of the albumin fusion, this strategy exploits the continuous recycling of albumin mediated by the neonatal Fc receptor (FcRn), which prevents endo-lysosomal degradation and is responsible for the exceptionally long circulation half-life of albumin of several days (Dixon et al., 1953; Peters, 1995). But an albumin-monobody fusion has a >6-fold higher molecular weight than a monobody alone and might therefore, like PEGylation or PASylation, not be compatible with cellular uptake strategies. Instead, various strategies caught our attention that focus on fusion to albumin binding peptides or albumin binding domains, which bind albumins from different species with high affinity. For example, an albumin-binding DARPin improved half-life of a fused target binder in both mice and monkeys (Steiner et al., 2017). But, as for albumin fusions, the molecular weight of a monobody-DARPin fusion would increase considerably and thereby hamper most intracellular delivery strategies. We also excluded serum albumin-binding antibody fragments for the same reason and due to the presence of disulfide bonds (Holt et al., 2008). On the other hand, a previously reported albumin-binding domain (ABD) derived from streptococcal protein G seemed to fulfill all our criteria (Stork et al., 2007). It is a highly soluble folded domain, only 56 amino acids long, and was shown to increase the half-life of an CEA + CD3-targeting bispecific single chain-diabody by 5-fold (Hopp et al., 2010). Therefore, it appeared to be well suited to be tested in the context of studying the pharmacological properties of monobodies, which have not been reported for the original monobody scaffold.

In this study, we show that monobodies have a high stability in plasma and retain their ability to bind their respective target protein *in vitro*. While no short-term toxicity was observed in mice, it was cleared by glomerular filtration *in vivo* within a few minutes. Therefore, we created and characterized bifunctional ABD-monobody fusions, that retained high affinity binding to its target and gained the ability to bind mouse and human albumin with low nanomolar affinities, while their molecular weight was only modestly increased. Not only did fusion with the ABD show a stabilizing effect on monobodies when incubated in mouse plasma over extended time periods, an ABD-monobody fusion also showed significantly enhanced pharmacological properties and biodistribution compared to its wildtype counterpart, following a single-dose intravenous injection in BALB/c mice: Circulation half-life was found to increase by 92-fold, which lead to a 265-fold greater area under the curve. Therefore, we demonstrate that the *in vivo* pharmacological properties and plasma stability of monobodies can be improved readily and efficiently through fusion with ABD, which represents an important step forward towards the development of monobodies to target intracellular oncogenes *in vivo*.

## 2 Materials and methods

### 2.1 Antibodies and reagents

Primary antibodies and detection reagents used for immunoblotting in this work: PentaHis-antibody (34660) and anti-AlexaFluor 488 antibody (A11094) were purchased from QIAGEN and Invitrogen, respectively. Primary antibodies were used at a 1:5,000 dilution in 5% BSA in Tris-Buffered Saline/0.1% Tween 20 (TBS-T) solution or 5% Milk/TBS-T solution according to manufacturer instructions. Secondary antibodies anti-mouse IRDye 680 (926–68072) and anti-rabbit IRDye 800 (926–32213) were obtained from LiCOR and used at a 1:10,000 dilution in TBS-T. IRDye 680-Streptavidin (926–68079) was purchased from LiCOR and used at 1:10,000 dilution in 5% Milk in Phosphate-Buffered Saline/0.1 % Tween 20 (PBS-T). AlexaFluor 488-Maleimide (APC-006) was purchased from Jena Bioscience. Non-sterile mouse plasma with sodium heparin (ABIN925342) was purchased from antibodies-online/Rockland Immunochemicals.

### 2.2 Plasmids and cloning

Amino acid sequence of Albumin Binding Domain 3 (ABD) has previously been reported (Stork et al., 2007). Gene Fragment encoding the Bcr-Abl SH2 domain-targeting monobody, AS25, fused to ABD (ABD-GGSGGGGSGG-AS25-Cys) was purchased from Twist Bioscience and cloned into a modified pET vector (Koide et al., 2007) containing a N-terminal 10xHis tag, FLAG tag and tobacco etch virus (TEV) protease cleavage site, using the restriction enzymes BamHI (R3436, New England Biolabs) and XhoI (R0146, New England Biolabs). Similarly, ABD-ML3-Cys was created through substitution of AS25 with the ML3 monobody (termed Mb (Lck_3), Kukenshoner et al., 2017), that targets the Lck-SH2 domain, using restriction enzymes NcoI (R3193, New England Biolabs) and XhoI. All DNA constructs were confirmed by DNA sequencing (Microsynth Seqlab, Göttingen, Germany). Full amino acid sequences of monobody constructs used in this work are reported in SI Supplementary Table S1.

### 2.3 Recombinant protein expression

Chemically competent BL21* cells (C601003, Life Technologies) were transformed by introduction of plasmids encoding recombinant proteins and plated on Agar plates supplemented with appropriate antibiotic. Single clones were used to inoculate pre-cultures in LB medium with appropriate antibiotic and incubated overnight at 37°C in a shaking incubator. Pre-cultures were transferred into Autoinduction medium (AIMLB0210, Formedium) with appropriate antibiotic and incubated at 37°C in a shaking incubator until OD600 reached ∼0.6–0.8, when cultures were transferred to 18°C and incubated overnight in a shaking incubator. Overnight expression cultures were spun-down at 4,000 × g and pellets resuspended in a Tris-based lysis/wash buffer (25 mM Tris-HCl pH 7.5, 300 mM NaCl, 5% Glycerol) and homogenized using an Avestin Emulsiflex C3 (Avestin, Ottawa, Canada). Cell lysates were spun-down for 45 min at 7,000 × g and supernatant loaded onto HisTrap FF crude columns (Cytiva, Amersham, United Kingdom), pre-equilibrated with wash buffer, in a Cytiva Äkta Avant system. The column was washed with wash buffer and monobody eluted with elution buffer (25 mM Tris-HCl pH 7.5, 300 mM NaCl, 5% Glycerol, 1 M Imidazole). Peak elution fractions were pooled und loaded onto HiLoad 16/600 Superdex 75 pg (Cytiva, Amersham, United Kingdom), pre-equilibrated with PBS, for preparative Size Exclusion Chromatography (SEC). Peak fractions corresponding to monomeric protein of interest were pooled, concentrated using Amicon Ultra Centrifugal Filters (Merck Millipore, Tullagreen, Ireland) and stored at −80°C until further use.

### 2.4 TEV protease cleavage

For TEV protease cleavage, proteins were incubated with in-house produced TEV protease at a ratio of 40:1 (protein:TEV protease, w/w) in PBS +0.5 mM EDTA and incubated for 4 h at room temperature under mild rotation. TEV protease, tags and TEV-cleaved protein of interest were separated using Size Exclusion Chromatography with a HiLoad 16/600 Superdex 75 pg as described above. Peak fractions containing the protein of interest were pooled, concentrated and stored at −80°C until further use.

### 2.5 AlexaFluor 488-labeling

Monobodies containing a C-terminal cysteine residue were mixed with AlexaFluor 488-Maleimide (AF488) at a molar ratio of 1:3 (monobody:AF488) and incubated protected from light for 3 h at room temperature under mild rotation. PD-MiniTrap G-25 columns (28918007, Cytiva) were equilibrated with PBS and excess dye removed from the labeling mix according to the manufacturer’s instruction. Protein concentration and degree of labeling (DOL) was measured at 280 nm (protein absorbance) and 495 nm (AF488 absorbance) absorbance with a NanoDrop 2000c (Thermofisher Scientific, Dreieich, Germany). Quality of labeled monobodies was checked by SDS-PAGE and analytical Size Exclusion Chromatography (aSEC) at 280 nm and 495 nm using a Superdex 75 10/300 GL (Cytiva, Amersham, United Kingdom).

### 2.6 Plasma stability assessment

Monobody stocks were prepared at a concentration of 50 µM in PBS. 50–75 µL of Stocks were subsequently mixed at a volume ratio of 1:1 with either mouse plasma (plasma) or PBS (control) and incubated at 37°C. At indicated time points, 20 µL samples were diluted 1:5 in PBS. 20 μL of this dilution were mixed with 7 µL of 4X Laemmli SDS buffer (400 mM DTT, 8% SDS, 200 mM Tris-HCl pH 6.8, 40% Glycerol, 0.02% Bromphenol blue) and denatured at 95°C for 5 min. These samples were separated using SDS-PAGE and blotted onto a 0.2 µm Amersham Protran nitrocellulose membrane (10600004, Cytiva). Monobodies were detected using either a murine penta-His antibody and anti-mouse IRDye 680 secondary antibody or a rabbit anti-AlexaFluor 488 antibody and anti-rabbit IRDye 800 secondary antibody. Additionally, samples were separated by SDS-PAGE and total protein stained using Coomassie Blue.

### 2.7 Target binding confirmation after plasma incubation

TEV-cleaved AlexaFluor 488-labeled monobodies AS25 and ML3 were prepared and mixed with mouse plasma as described above and incubated for 24 h at 37°C. Subsequently, AS25 was either mixed with an equimolar amount of biotinylated Bcr-Abl SH2 domain (Abl-SH2) or PBS, incubated for 1 h at room temperature under mild shaking and analyzed by SEC on a Superdex 75 Increase 10/300 GL column. Absorbance at 280 nm and 495 nm was monitored. Abl-SH2 alone was eluted without prior incubation in plasma. Similarly, ML3 was either mixed with an equimolar amount of biotinylated LCK-SH2 domain or PBS, incubated for 1 h at room temperature under mild shaking and analyzed by SEC on a Superdex 75 Increase 10/300 GL column. LCK-SH2 alone was eluted without prior incubation in plasma. For detection of complex formation, fractions were analyzed by SDS-PAGE and blotted onto a 0.2 µm nitrocellulose membrane as described above. An anti-AlexaFluor 488 antibody was used overnight at 4°C, before the membrane was washed in TBS-T, followed by a 1:1 PBS-T/TBS-T mix and PBS-T. Afterwards, the membrane was incubated with anti-rabbit IRDye 800 and IRDye 680-Strepatividin for 1 h at room temperature.

### 2.8 Isothermal titration calorimetry (ITC)

Recombinant proteins were extensively dialyzed overnight at 4°C into PBS and were degassed. Total protein concentration was measured at 280 nm with a NanoDrop 2000c. ITC measurements were performed as previously described using a MicroCal PEAQ ITC (Malvern Panalytical, Kassel, Germany) and consisted of 19 titration steps from the syringe to the cell, with a first injection of 0.4 µL followed by 18 injections of 2.0 µL and a spacing of 150 s between injections (Schmidt et al., 2022). Protein concentrations were set to a ratio of 10:1 (syringe:cell) as indicated in the figure legends. The MicroCal software was used to determine thermodynamic parameters, including dissociation constant (*K*_*d*_), enthalpy (*ΔH*) and binding stoichiometry (*N*).

### 2.9 Short-term toxicity assessment in BALB/c mice

Female BALB/c mice (6-7 weeks, 16–19 g at Day 1) were assigned into nine groups of 3 animals each and received a single 100 µL intravenous injection into the tail vein on Day 1. Group 1 received vehicle control (PBS). Groups 2–5 received monobody ML3 at 1, 3, 5 or 10 mg/kg, respectively. Groups 6–9 were injected with monobody AS25 at 1, 3, 5 or 10 mg/kg, respectively. Prior to, during and up to 72 h after injection, health, behavior and body weight of the animals were regularly observed by a qualified veterinarian. Injection and supervision were done by a contract research organization following our study protocol (Creative Biolabs, Shirley, United States).

### 2.10 ^125^I-Radiolabeling of monobodies

AS25 or ABD-AS25, containing a C-terminal cysteine residue, were radiolabeled with Iodine-125 using a radioactive thiol reactive maleimide, N-[2-(maleimido) ethyl]-3-iodo-benzamide (^125^I-BM). Briefly, ∼0.3 nmol of ^125^I-BM solution in dimethylformamide were mixed with ∼500 µg of intact monobody in PBS and incubated for 30 min at room temperature. Unreacted ^125^I-BM was then removed by a PD-10 desalting columns equilibrated in 10 mM PBS/2 mM EDTA/0.1% Tween 80. Radiochemical purity and total activity of the labeled monobody-solution were determined by instant Thin Layer Chromatography (iTLC, 10% trichloroacetic acid in water) and gamma counting (Wizard2 2470, Perkin Elmer), respectively. Protein concentration was determined by absorbance at 280 nm and the specific activity in mCi/mg was calculated. A dosing solution at 0.25 mCi/mg specific activity and 1 mg/mL concentration was then prepared by isotopic dilution with non-labeled monobody. Sufficient radiolabeling and stability of monobodies were confirmed by SEC, SDS-PAGE and autoradiography. Detailed radiolabeling characteristics and results are reported in SI. Radiolabeling was done by a contract research organization (Chelatec SA, Saint-Herblain, France).

### 2.11 Pharmacokinetics and biodistribution assessment of AS25 in BALB/c mice

A total of 12 female BALB/c mice (9 weeks old, 23–26 g at Day 1) received a single intravenous injection of radiolabeled AS25 (AS25-^125^I) at 5 mg/kg into the retro-orbital plexus. At intermediate timepoints of 2, 5, 10, and 20 min after injection, blood of 3 mice per each time point was sampled from the retro-orbital plexus. At terminal time points of 15, 30, 45, and 60 min after injection, 3 mice per each time point were sacrificed and blood samples obtained from exsanguination. Radioactivity of blood samples and plasma samples, prepared by centrifugation of blood samples, was measured by gamma-counting. Levels of AS25 were calculated as percentage of injected dose per mL of blood or plasma (%ID/mL) and total volume of blood or plasma (%ID). Additionally, selected tissues/organs (liver, thymus, kidneys, spleen, lungs, heart, intestinal tract, thyroid) were harvested of 3 mice per each terminal time point and radioactivity analyzed by gamma-counting. Levels of AS25 were calculated as percentage of injected dose per gram of organ/tissue (%ID/g) and whole organ (%ID). These experiments were done by a contract research organization following our study protocol (Chelatec SA, Saint-Herblain, France) and were approved by the ministry of higher education, research and innovation France (file reference APAFIS#27745-2015120110381211 v4). The study was conducted in accordance with the local legislation and institutional requirements.

### 2.12 Pharmacokinetics and biodistribution assessment of ABD-AS25 in BALB/c mice

A total of 18 female BALB/c mice (8 weeks old, 20–23 g at Day 1) received a single intravenous injection of radiolabeled ABD-AS25 (ABD-AS25-^125^I) at 5 mg/kg into the retro-orbital plexus. At intermediate timepoints of 2 min, 5 min, 10 min, 20 min, 45 min, and 3 h after injection, blood of 3 mice per each time point was sampled from the retro-orbital plexus. At terminal time points of 15 min, 30 min, 60 min, 6 h, 14 h, and 24 h after injection, 3 mice per each time point were sacrificed and blood samples obtained from exsanguination. Radioactivity of blood samples and plasma samples, prepared by centrifugation of blood samples, was measured by gamma-counting. Levels of ABD-AS25 were calculated as percentage of injected dose per mL of blood or plasma (%ID/mL) and total volume of blood or plasma (%ID). Additionally, selected tissues/organs (liver, kidneys, lungs, heart, bladder) were harvested of 3 mice per each terminal time point and radioactivity analyzed by gamma-counting. Levels of ABD-AS25 were calculated as percentage of injected dose per gram of organ/tissue (%ID/g) and whole organ (%ID). These experiments were done by a contract research organization following our study protocol (Chelatec SA, Saint-Herblain, France) and were approved by the ministry of higher education, research and innovation France (file reference APAFIS#27745-2015120110381211 v4). The study was conducted in accordance with the local legislation and institutional requirements.

## 3 Results

### 3.1 Monobodies are stable in mouse plasma

**FIGURE 1:**
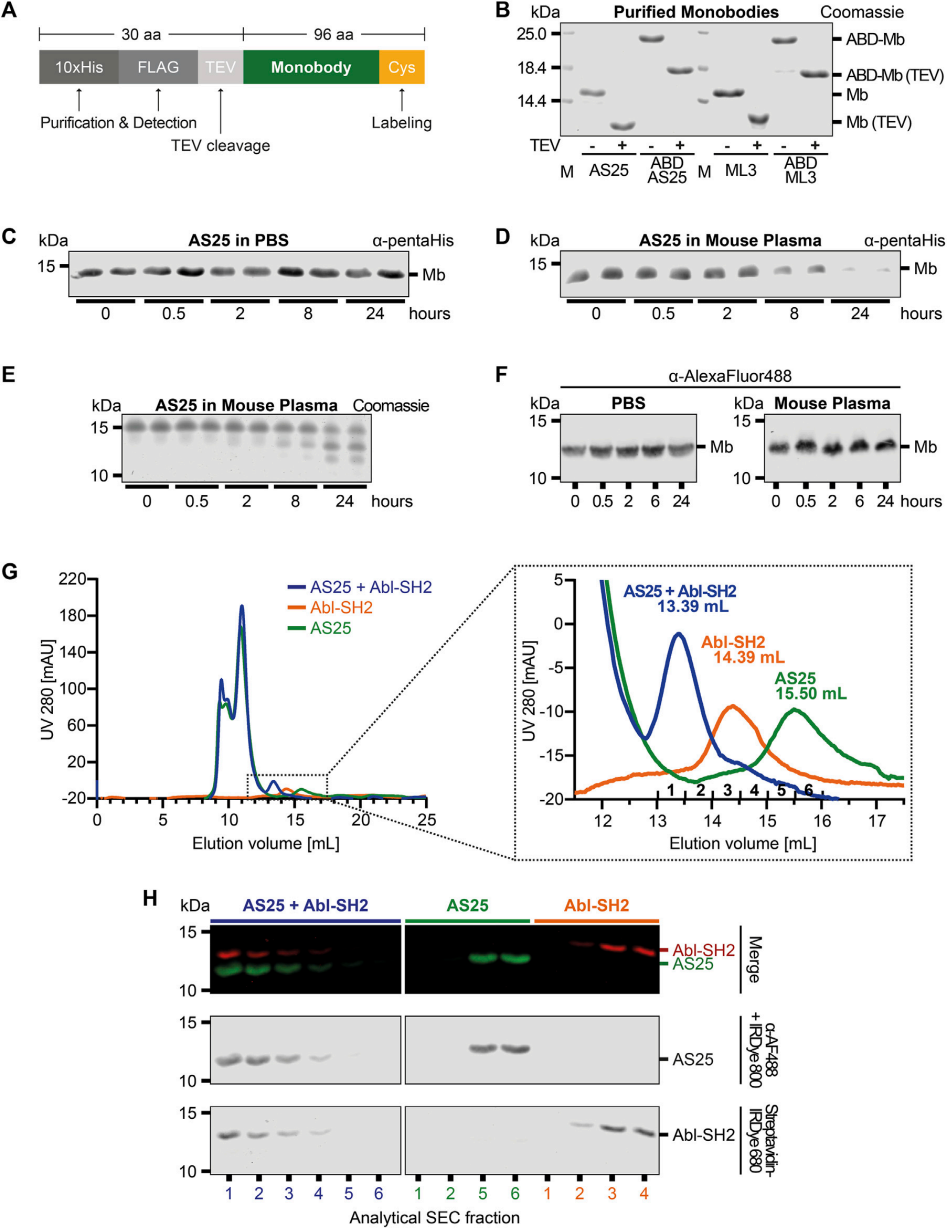
Plasma Stability and Target Binding of Monobody AS25 **(A)** Schematic representation of the expression constructs used for recombinant expression in *E. coli* and subsequent purification of monobodies. An N-terminal 10xHis and a FLAG-tag is used for detection or purification. A TEV protease cleavage site and a C-terminal cysteine residue are used for removal of the 10xHis-FLAG tags and covalent labeling of monobodies, respectively. **(B)** SDS-PAGE analysis of monobody constructs expressed in *E. coli* and purified by Ni-affinity chromatography and size exclusion chromatography. After purification, N-terminal tags were cleaved from indicated samples using TEV protease. TEV-cleaved proteins were separated from uncleaved proteins and TEV protease using size exclusion chromatography. 4 μg of purified monobody proteins were separated using SDS-PAGE. Total protein was stained using Coomassie Blue. **(C,D)** Plasma stability analysis of uncleaved AS25 (Mb). The monobody was incubated in PBS **(C)** or mouse plasma **(D)** at 37°C. Samples were taken from biological duplicates at indicated timepoints and analyzed by SDS-PAGE and immunoblotting. Levels of monobody were assessed by an anti-pentaHistidine antibody. Representative immunoblots from three to six repeats are shown. **(E)** Samples from panel C were analyzed by total protein staining using Coomassie Blue. **(F)** Plasma stability analysis of TEV-cleaved AS25. Purification tags were removed from AS25 using TEV protease cleavage and the monobody was C-terminally labeled with AlexaFluor 488 before incubation in either PBS or mouse plasma. Samples were taken at indicated timepoints and analyzed by SDS-PAGE and immunoblotting. Levels of monobody were assessed by an anti-AlexaFluor 488 antibody. Representative immunoblots from three to six repeats are shown. **(G)** Analytical size exclusion chromatography analysis of complex formation of TEV-cleaved AS25 with its target, Abl-SH2, after incubation in mouse plasma. AS25 was either mixed with equimolar concentrations of Abl-SH2 (blue) or PBS (green) and eluted from a Superdex 75 Increase 10/300 GL column. Abl-SH2 alone was eluted without prior incubation in plasma (orange). The dotted rectangle in the left chromatogram indicates area shown as close-up on the right. mAU: milli-absorbance units. **(H)** Immunoblot of fractions sampled from analytical SEC from **(G)**. AlexaFluor 488-labeled AS25 and biotinylated Abl-SH2 domain were detected by an anti-AlexaFluor 488 antibody + IRDye800 secondary antibody (middle panel) and Streptavidine-IRDye680 (lower panel). Merge shows both channels combined (upper panel, red = Abl-SH2, green = AS25).

We measured plasma stability of recombinantly expressed monobodies, as a first approximation for *in vivo* stability. Monobodies AS25 and ML3, targeting the Bcr-Abl and Lck SH2 domain, respectively, were expressed with an N-terminal 10xHis-Flag-tag and a TEV protease cleavage site (Figures 1A, B; (Wojcik et al., 2016; Kukenshoner et al., 2017). The purified monobodies were incubated in mouse plasma or PBS and detected by immunoblotting with antibodies recognizing the 10xHis-Flag-tag purification tags. After 8 h of incubation in plasma, a strongly decreased signal was detected. At 24 h, close to no signal was detectable anymore (Figures 1C, D; Supplementary Figure S7). In contrast, no signal decrease was observed in PBS (Figures 1C, D; Supplementary Figure S7). Analysis of these samples by Coomassie Staining revealed bands of smaller molecular weight than the full-length monobody at 8 and 24 h (Figure 1E). Mass spectrometry analysis showed that the mass of these bands is in line with cleavage of the 10xHis-Flag-purification tags while leaving the monobody intact (data not shown). To overcome these shortcomings in monobody detection, we removed the purification tags by TEV-protease cleavage and labeled the AS25 and ML3 monobodies with an AlexaFluor488-dye at a cysteine residue that was engineered as the C-terminal residue after the last β-strand of the monobody scaffold. Using this modified monobody preparation, no significant decrease in signal or molecular weight of TEV-cleaved monobodies was observed at any tested timepoint, indicating a high stability of monobodies for at least 24 h in mouse plasma (Figure 1F; Supplementary Figure S1, S7).

### 3.2 Monobodies retain target binding in plasma

Next, we sought to test whether monobodies retain their folding and function in plasma. Hence, we tested whether monobodies are still able to bind their target proteins after plasma incubation. We incubated TEV-cleaved AlexaFluor 488-labeled AS25 in mouse plasma for 24 h and added an equimolar amount of its target, the Bcr-Abl SH2 domain (Abl-SH2) carrying a biotin for detection. A peak with smaller elution volume (fractions 1 and 2) was detected by analytical size exclusion chromatography (aSEC) in samples containing both AS25 monobody and Abl-SH2 at concentration above the *K*_*d*_ of the interaction. In contrast, samples containing AS25 or Bcr-Abl SH2 domain alone eluted later (Figure 1G). This indicates that monobodies retain target binding after plasma incubation and that target binding is not perturbed by plasma proteins. Immunoblotting analysis confirmed efficient monobody-target complex formation, as both proteins co-eluted and were detected in fractions 1 and 2 and no signal corresponding to unbound AS25 or Abl SH2 was detected in later fractions. In the samples containing the individual proteins, Bcr-Abl SH2 and AS25 were detected in fractions 4-5 and 5-6, respectively (Figure 1H). Similar results were obtained for the ML3 monobody (Supplementary Figure S1). Therefore, it seems that no cleavage or post-translational modification events of the monobodies happen in plasma that would perturb monobody folding or binding to its target proteins. Collectively, these results show that monobodies are stable and able to bind their targets in mouse plasma.

### 3.3 Monobodies show no short-term toxicity in BALB/c mice

For *in vivo* application of recombinant proteins produced in *E. coli*, it is important to ensure lack of endotoxins, which can trigger immune responses leading to inflammation and possible serious adverse events, thereby impinging on the safety and efficacy of biopharmaceutical products. Therefore, we measured endotoxin levels of our monobody preparations using the limulus amebocyte lysate (LAL) test. After purification of monobodies by affinity chromatography followed by size exclusion chromatography, a high purity (>95%) of monobodies was achieved (see Figure 1B) and the LAL test showed low endotoxin levels, below the tolerable threshold of 1.5 Units/mL (Malyala and Singh, 2008). Hence, we next intravenously injected monobodies at four different concentrations (1–10 mg/kg) into BALB/c mice and observed the animals for 72 h. No mortality, moribundity or abnormalities in general health status, behavior, skin, hair, feces, urine or other abnormal reactions were observed. In addition, no monobody-related weight change was observed, compared to the vehicle control group (Supplementary Figure S2; Supplementary Table S2).

### 3.4 Monobodies have a short half-life in mice

**FIGURE 2:**
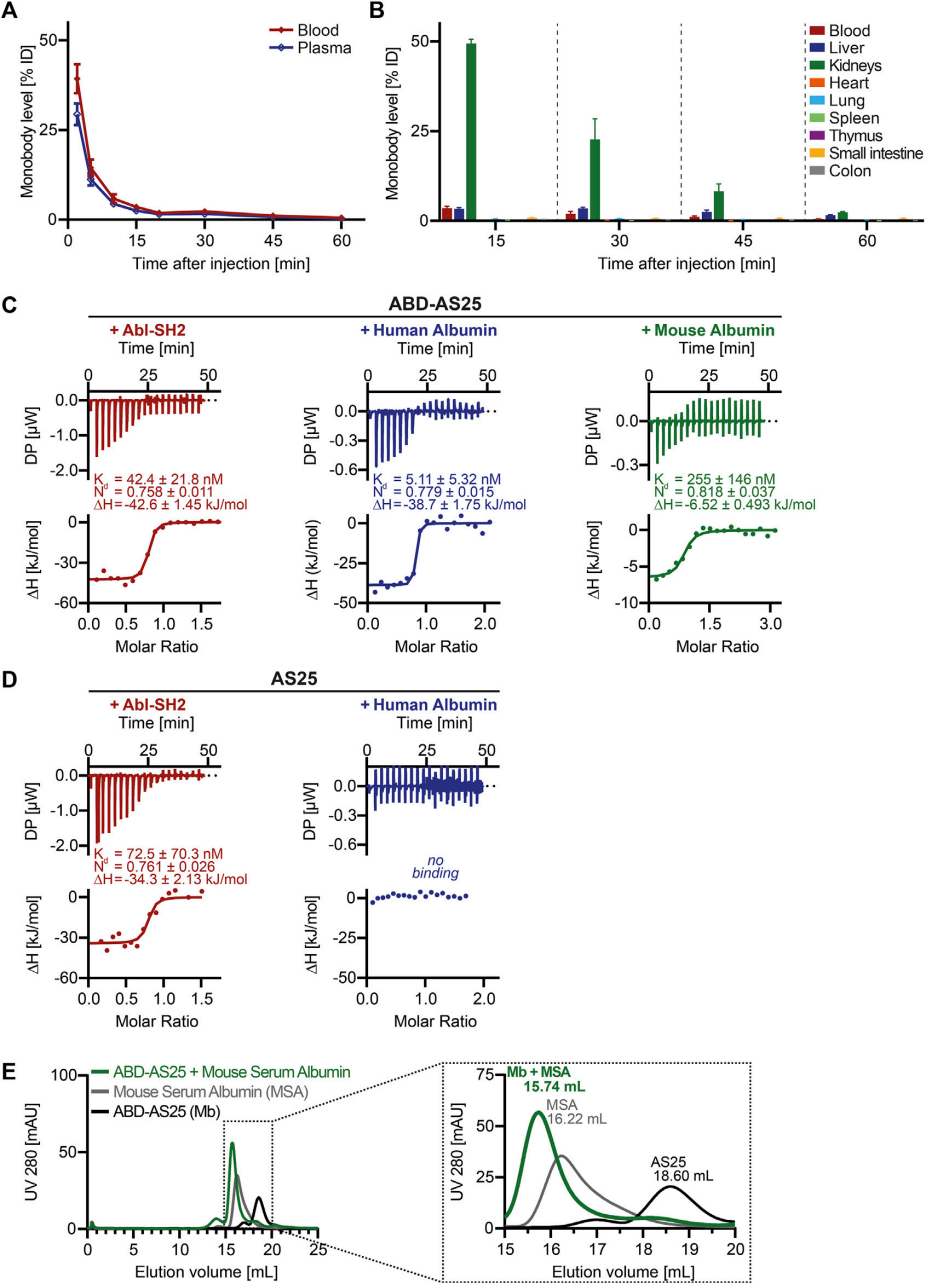
Pharmacokinetics of AS25 in mice and development of Albumin Binding Monobody ABD-AS25 **(A)** Blood (red line) and plasma (blue line) levels of Iodine-125 labeled monobody AS25 in BALB/c mice after single i.v.-injection at 5 mg/kg. Samples were taken at the indicated timepoints from three individual mice each and monobody quantified by gamma-counting. Monobody levels were calculated as percentage of injected dose per total volume of blood or plasma (%ID) and are plotted as *Mean ± SD*, *n = 3*. **(B)** Biodistribution of Iodine-125 labeled Monobody AS25 in BALB/c mice after single i.v.-injection at 5 mg/kg. Organs were harvested from three individual mice at the indicated timepoints, rinsed in physiological serum, weighed and monobody quantified by gamma-counting. Monobody levels were calculated as percentage of injected dose per whole organ (%ID). Monobody levels are shown as *Mean ± SD*, *n = 3.* **(C,D)** Isothermal titration calorimetry (ITC) measurements of ABD-AS25 and AS25 with Abl-SH2 (red), Human Albumin (blue) or Mouse Albumin (green) at 25°C Each panel shows the raw heat signal of a representative ITC experiment (top) and the integrated calorimetric data of the area of each peak (bottom). The continuous line represents the best fit of the data computed from the MicroCal software. Dissociation constant (*K*_*d*_), enthalpy (*ΔH*) and stoichiometry (*N*) are calculated from the fit. **(C)** *red*: Abl-SH2 (190 µM) titrated to ABD-AS25 (20 µM), *blue*: Human Serum Albumin (105 µM) titrated to ABD-AS25 (10 µM), *green*: ABD-AS25 (196 µM) titrated to Mouse Serum Albumin (12 µM). **(D)** *red*: Abl-SH2 (192 µM) titrated to AS25 (24 µM), *blue*: Human Serum Albumin (103 µM) titrated to AS25 (4.5 µM). **(E)** Analytical size exclusion chromatography analysis of complex formation of ABD-AS25 with mouse serum albumin. ABD-AS25 was either mixed with equimolar amounts of mouse serum albumin (green) or PBS (black) and eluted from a Superdex 200 Increase 10/300 GL column. Mouse serum albumin alone was run as additional control (grey). The dotted rectangle in the left chromatogram indicates area shown as close-up on the right. *mAU*: milli-absorbance units.

Given that monobodies show no short-term toxicity, we next determined pharmacokinetics and biodistribution in mice. We prepared an Iodine-125 labeled AS25 monobody at high specific activity (9.40 MBq/mg). AS25-^125^I was purified by SEC and eluted as a single monomeric peak and no degradation or impurities were visible in SDS-PAGE and autoradiography (Supplementary Figure S3). Incubation of this monobody for 48 h in PBS or 24 h in mouse plasma showed >95% retention of the ^125^I-label and lack of oligomerization/aggregation (data not shown). Next, we intravenously injected the monobody at 5 mg/kg into BALB/c mice. A rapid decline of monobody levels was observed in blood and plasma. (Figure 2A). Less than 40% of injected dose could be detected in blood after 2 min, resulting in a circulation half-life of less than 2 min and area under the curve (AUC) of only ∼2.5 µg/(mL × h) (Figure 2A). In addition, we analyzed monobody levels in eight internal organs and blood. The majority of the injected monobody (∼50%) was found in the kidneys after 15 min and halved every other 15 min. In blood and liver only ∼3.5% were detected. All other organs showed very low levels (<1%). By 60 min, no monobody was detectable in any organ (Figure 2B). These results indicated unfavorable pharmacokinetics of monobodies, including an unfavorable organ distribution and rapid excretion, for a possible future *in vivo* therapeutic use.

### 3.5 Development of albumin binding domain-monobody fusions

We next surveyed different strategies to improve *in vivo* half-life of biopharmaceutics and evaluated their applicability to monobodies. While several approaches, including PEGylation and Albumin fusion were deprioritized due to concerns of compatibility with intracellular monobody delivery approaches, we focused on albumin binding domains (ABDs). Therefore, we fused a 56 amino acid ABD to the N-terminus of monobodies (Stork et al., 2007). Including linkers and purification tags, molecular weight was only modestly increased to ∼21 kDa (Figure 1B). These ABD-monobodies were highly soluble in *E.coli* and could be purified with high yield and purity (Figure 1B). ABD-AS25 monobody was monomeric in SEC and was subsequently assayed for ligand binding using isothermal titration calorimetry (ITC). ABD-AS25 bound the Abl SH2 domain with a dissociation constant (*K*_*d*_) of 42.4 nM. Human and mouse albumin were also bound with high affinity (5.11 nM and 255 nM, respectively; Figure 2C). All ITC measurements suggested a binding stoichiometry of 1:1 (Figure 2C). While AS25 (without ABD) did bind Abl SH2 with similar affinity, no binding to human albumin was observed (Figure 2D). In addition, ABD-AS25 showed efficient complex formation with mouse serum albumin in aSEC (Figure 2E). These data showed that bispecific ABD-monobody fusions do not affect monobody-target interaction and enable high affinity binding to albumin.

### 3.6 High plasma stability of ABD-AS25

**FIGURE 3:**
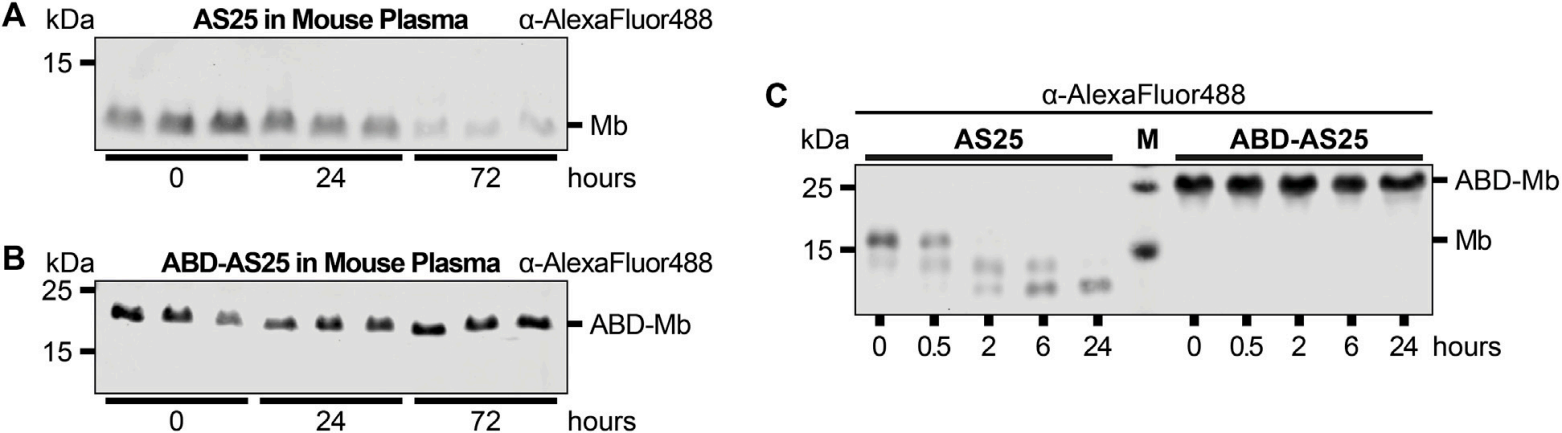
Plasma Stability comparison of AS25 and ABD-AS25 **(A,B)** Representative immunoblots of the plasma stability of TEV-cleaved AS25 (A, Mb) and TEV-cleaved ABD-AS25 (B, ABD-Mb). Purification tags were removed from AS25 and ABD-AS25 using TEV protease cleavage and the monobodies were C-terminally labeled with AlexaFluor 488 before incubation in mouse plasma. Samples were taken at the indicated timepoints and analyzed by SDS-PAGE and immunoblotting. Levels of monobody were determined by an anti-AlexaFluor 488 antibody. **(C)** Representative immunoblot of plasma stability comparison of uncleaved AS25 (Mb) and uncleaved ABD-AS25 (ABD-Mb). The monobodies were incubated in mouse plasma at 37°C. Samples were taken at indicated timepoints and analyzed by SDS-PAGE and immunoblotting. Levels of monobody were determined by an anti-AlexaFluor 488 antibody.

We next studied the plasma stability of ABD-AS25. As for TEV-cleaved AS25, no degradation of TEV-cleaved ABD-AS25 was observed in the first 24 h of plasma incubation. After extended incubation of up to 72 h, no appreciable degradation of TEV-cleaved ABD-AS25 was detected, whereas TEV-cleaved AS25 levels decreased to less than 25% of the initial amount (Figures 3A, B; Supplementary Figure S7). Surprisingly, uncleaved ABD-AS25, which still contained its purification tags, did not show the same degradation pattern as uncleaved AS25 described above (see Figures 1E, 3C). Parallel evaluation of an ABD-ML3 monobody fusion confirmed higher plasma stability and lack of tag degradation (Supplementary Figure S4). Taken together, fusion of monobodies to an ABD increased plasma stability, possibly by shielding monobodies from proteolytic degradation through albumin binding.

### 3.7 ABD-AS25 shows improved pharmacokinetics

**FIGURE 4:**
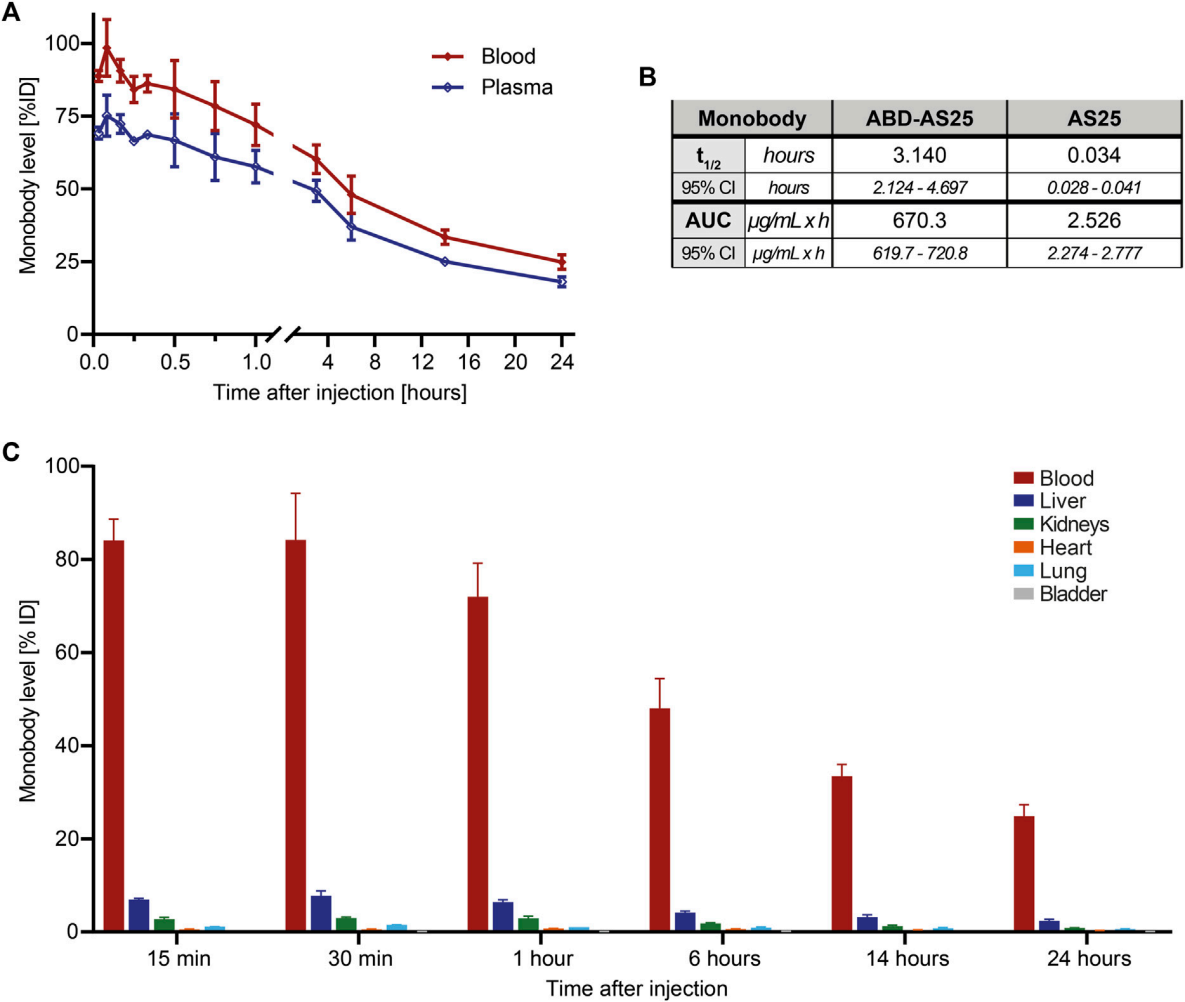
Pharmacokinetics of ABD-AS25 in BALB/c mice **(A)** Blood (red line) and plasma (blue line) levels of Iodine-125 labeled monobody ABD-AS25 in BALB/c mice after single i.v.-injection at 5 mg/kg. Samples were taken at the indicated timepoints from three individual mice each and assessed by gamma-counting. Monobody levels were calculated as percentage of injected dose per total volume of blood or plasma (%ID) and are plotted as *Mean ± SD*, *n = 3*. **(B)** Comparison of circulation half-life (*t*_*1/2*_) and area under the curve (*AUC*) for ABD-AS25 and AS25. Values were calculated using data of blood levels from panel 2A and 4A, using GraphPad Prism functions *Half-life (One Phase)* and *AUC*. Half-life is presented as mean from best fit with 95% confidence interval (95% CI). AUC is reported as total peak area with 95% confidence interval (95% CI). **(C)** Biodistribution of Iodine-125 labeled Monobody ABD-AS25 in BALB/c mice after single i.v.-injection at 5 mg/kg. Organs were harvested from three individual mice at the indicated timepoints, rinsed in physiological serum, weighed and monobody levels quantified by gamma-counting. Monobody levels were calculated as percentage of injected dose per whole organ (%ID) and are plotted as *Mean ± SD*, *n = 3*.

We next labeled ABD-AS25 with Iodine-125, as described above, which showed high stability in PBS and mouse plasma and lacked oligomerization/aggregation (Supplementary Figure S5). We injected ABD-AS25-^125^I intravenously at 5 mg/kg into BALB/c mice and observed monobody levels in blood, plasma and selected organs for up to 24 h. Monobody levels showed a shallow decline over time, with >70% remaining in blood at one hour, ∼33% at 14 h and ∼25% at 24 h after injection (Figure 4A). These levels correspond to a circulation half-life of 3.14 h (∼188 min) and an AUC of 670.3 µg/(mL × h). Therefore, inclusion of the ABD increased the half-life of AS25 by 92-fold and AUC by 265-fold (Figure 4B; Supplementary Figure S8). We measured monobody levels in different internal organs to understand its distribution and excretion. In contrast to AS25, >80% of ABD-AS25 was found to remain in blood at 15 min post-injection, whereas ∼7.5% and ∼3% were detectable in liver and kidneys, respectively (Figure 4C). All other organs showed very low levels (<1%) of ABD-AS25. Over time, no appreciable organ accumulation was found and monobody levels declined gradually in all organs (Figure 4C). In summary, fusion of ABD to monobodies strongly increased circulation half-life without causing unwanted organ accumulation. Therefore, this approach represents a simple and versatile method to boost further development of monobodies for *in vivo* application.

## 4 Discussion

Our work described here, provides primary insight into the stability and pharmacokinetics of monobodies and reports a facile method to enhance not only their pharmacological properties but also the stability of monobodies to overcome initial shortcomings. While the initial assessment of *in vivo* pharmacokinetics of the AS25 monobody revealed a rapid clearance from the bloodstream and presence of high levels of monobody in the kidneys, fusion of a small albumin biding domain to the monobody led to a 92-fold and 265-fold increase in half-life and AUC, respectively, emphasizing the versatility and promise of monobodies to be modified and developed from basic research tools into future therapeutic candidates.

While our assessment of plasma stability showed the N-terminal purification tags of the monobody to be vulnerable to rapid degradation, the core beta-sandwich fold of the FN3 scaffold remained stable in mouse plasma, in line with previous reports on the biophysical stability of monobodies *in vitro*. Also, our mass spectrometry data may indicate that there are no major post-translational modifications of monobodies in plasma, which would impede target binding. This is supported by the finding that efficient and unperturbed target interaction after incubation, even in the complex and crowded environment of mouse plasma was detected. Surprisingly, ABD-monobody fusions of two different monobodies (ABD-AS25 and ABD-ML3) showed little degradation over 72 h, whereas their non-fusion counterparts showed considerable degradation. To our knowledge, this effect has not been previously reported. One could envision that binding to albumin may possibly impede degradation by hindering binding of plasma proteases.

**FIGURE 5:**
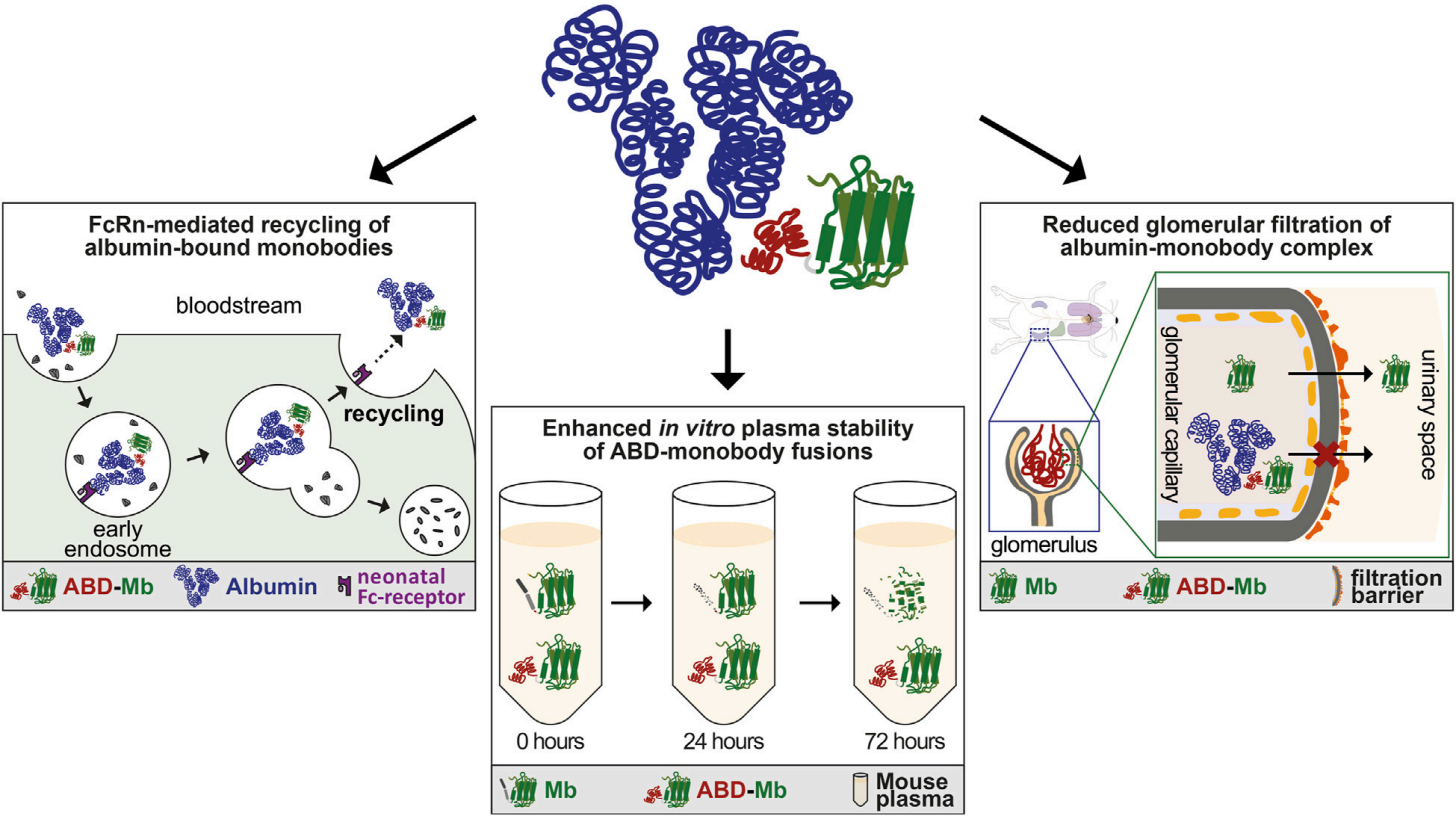
Mechanisms influencing enhanced pharmacokinetics of ABD-monobody fusions. Schematic overview of possible mechanisms leading to enhanced pharmacokinetics of ABD-monobodies. Upper mid panel: Cartoon of ABD-monobody in complex with albumin. The albumin binding domain (red) fused to the monobody core (green) binds to domain II of albumin (blue). Left panel: ABD-monobodies exploit the FcRn-mediated recycling pathway through binding to albumin, protecting them from endosomal degradation and leading to recycling to the bloodstream. Lower mid panel: ABD-fusions show increased stability *in vitro* over longer periods of incubation in mouse plasma, which might be predictive to stability *in vivo*. Right panel: The increased size and hydrodynamic radius of the ABD-monobody-albumin complex prevents glomerular filtration in the kidneys.

The rapid clearance of unmodified monobodies *in vivo* was due to loss of protein from circulation through renal filtration, commonly reported for proteins of similar size. On one hand, this is clearly a disadvantage of small protein therapeutics, where a high plasma concentration and slow clearance is desirable for sufficient therapeutic effect, as otherwise more frequent dosing or continuous infusion would be needed. On the other hand, unmodified monobody variants were used as PET-tracers, for which rapid clearance is favored (Donnelly et al., 2018). The strong half-life prolonging effect of the ABD that we observed, might be due to three different, possibly interconnected mechanisms. Firstly, the binding to albumin enables ABD-monobody fusions to piggyback on the neonatal Fc-receptor (FcRn-)mediated recycling of albumin (Figure 5). This mechanism is widely reported to be responsible for the exceptionally long circulation half-life of human and mouse albumin and exploited by several recombinant protein drugs (Chaudhury et al., 2003; Metzner et al., 2013). Although its name is based on the transport of maternal IgG across the placenta to passively immunize the fetus, FcRn is also widely expressed in adult tissues, including the vascular endothelium, liver, spleen and kidney (Sarav et al., 2009). In the kidneys, where it is located on the podocytes and the brush border of the proximal tubular epithelial cells, it is able to reclaim albumin to maintain serum levels (Sarav et al., 2009). Secondly, the higher plasma stability of ABD-fused monobodies *in vitro* may also translate to increased plasma stability *in vivo*, as ABD-monobodies might be less susceptible to proteolytic degradation in the bloodstream (Figure 5). Thirdly, fusion of the ABD to the monobodies not only mildly increases the size of the resulting ABD-fusion (∼20 kDa), but also results in complex formation with albumin, leading to a complex with significantly increased size (∼90 kDa) and hydrodynamic radius, that is larger than the renal filtration threshold (Figure 5).

Since ABD-monobodies could be detected for extended times in the bloodstream, this enables their exposure to different target organs and tumors. On the other hand, this did not result in accumulation in specific organs, particularly liver or kidneys, which could otherwise lead to concerns of organ toxicity. High monobody plasma levels are also an important prerequisite for the combination of half-life extension with cellular delivery approaches for monobody proteins that we are exploring to enable intracellular targeting of oncogenes (Hantschel, 2017; Schmit et al., 2019).

While we did not observe any short-term toxicity or acute immune reaction of up to 10 mg/kg of monobodies in mice, observations of this study were limited to a single dose injection. Hence, possible effects of long-term and multiple dosing remain to be determined. In particular, the emergence of an antibody response to the protein therapeutic, which is commonly observed even for fully humanized antibody therapeutics, is of concern (Harding et al., 2010). Because monobodies are engineered on an FN3 domain scaffold of human fibronectin and all monobodies have only ∼20 mutated amino acids spread across different loops and beta-strands to enable target binding, one can assume that there is only a low chance of strong immunogenicity, unless an immunodominant peptide is generated. To counteract immunogenicity and further enhance stability, we are developing mirror-image monobodies that are composed of D-amino acids (Schmidt et al., 2022; Hantschel lab, unpublished observations).

Since we aim to target hitherto undruggable intracellular oncoproteins with monobodies, delivery into tumor cells remains a key challenge, which we currently address using different strategies (Schmit et al., 2019; Feng et al., 2023). A smaller cargo protein size is generally preferable to achieve high cellular delivery efficiency. Hence, for improving *in vivo* half-life and stability, methods that significantly increase monobody size were deprioritized, and we focused on the ABD, which limits the size of the ABD-monobody fusion to ∼20 kDa. To test the compatibility of ABD-fusion with cellular delivery, we performed preliminary experiments with “supercharged” monobodies, which are taken up in cancer cells. We observed that the fusion with the ABD only mildly impaired cellular uptake of “supercharged” monobodies in media with and without albumin (Hantschel lab, unpublished observations) This indicates that the ABD used in this work can be combined with future approaches aiming at delivering functional monobodies to the cytoplasm of cancer cells.

In this work, we have demonstrated the improvement of pharmacokinetics, biodistribution and stability of monobodies through fusion with an albumin binding-domain, creating ABD-monobodies that retained target binding and were able to bind albumin with high affinity. This demonstrated the high modularity and adaptability of monobodies for different fusion partners without loss of binding of the individual modules. Given that the parental fibronectin is build-up from multiple fibronectin-domains like “beads on a string,” we previously created tandem monobodies, which have been employed to bind two different target interfaces of the Bcr-Abl SH2 domain at the same time or to use tandem monobodies with specificity for two different targets as glues to induce protein-protein interactions (Grebien et al., 2011; Hantschel lab, unpublished results). In addition, we are currently exploring strategies to increase tumor cell-selectivity of monobodies by fusion with FN3-based tumor-targeting moieties, that bind PD-L1 or EGFR (Hackel et al., 2012; Donnelly et al., 2018; Hantschel lab, unpublished results). Alternative binders to other cell-surface markers, such as DARPins for glutamate receptor subunit GluA4, the endothelial surface marker CD105, and the natural killer cell marker NKp46 were developed for gene- and cell-therapy approaches and can be used to target monobodies to specific cell types (Hartmann et al., 2018). Still, it needs to be carefully assessed, how the interplay between the different binding moieties will influence cell binding, cellular uptake and function of the resulting monobodies.

## 5 Conclusion

In conclusion, our results present an efficient method of enhancing the previously unfavorable *in vivo* properties of monobodies, resulting in superior pharmacokinetics, biodistribution and stability without impeding binding capability, emphasizing the flexibility and versatility of modifications that can be applied to monobodies. The insights gained from our studies position monobodies as promising candidates to be developed into safe, effective and specific protein therapeutics to extend the armamentarium to fight cancer.

## Data Availability

The original contributions presented in the study are included in the article/Supplementary Material, further inquiries can be directed to the corresponding author.
