# Supplementary material for: Improving the pharmacokinetics, biodistribution and plasma stability of monobodies
Source: Front Pharmacol. 2024 Apr 4;15:1393112. doi: 10.3389/fphar.2024.1393112 (PMC7615827; doi:10.3389/fphar.2024.1393112)
Supplement: Supplementary file 1 [file DataSheet1.PDF]

Supplementary Material

1 Supplementary Data

None.

2 Supplementary Figures and Tables

2.1 Supplementary Figures

Supplementary Figure 1. Plasma Stability and Target Binding of Monobody ML3

Supplementary Figure 2. Body weights of BALB/c mice injected with Monobodies

Supplementary Figure 3. Quality control of unlabeled and Iodine-125 labeled AS25

Supplementary Figure 4. Plasma Stability comparison of ML3 and ABD-ML3

Supplementary Figure 5. Quality control of unlabeled and Iodine-125 labeled ABD-AS25

Supplementary Figure 6. Uncropped SDS-PAGE and immunoblot images used in this work

Supplementary Figure 7. Statistical analysis of plasma stability of monobodies

Supplementary Figure 8. Statistical analysis of blood levels of monobodies in BALB/c mice

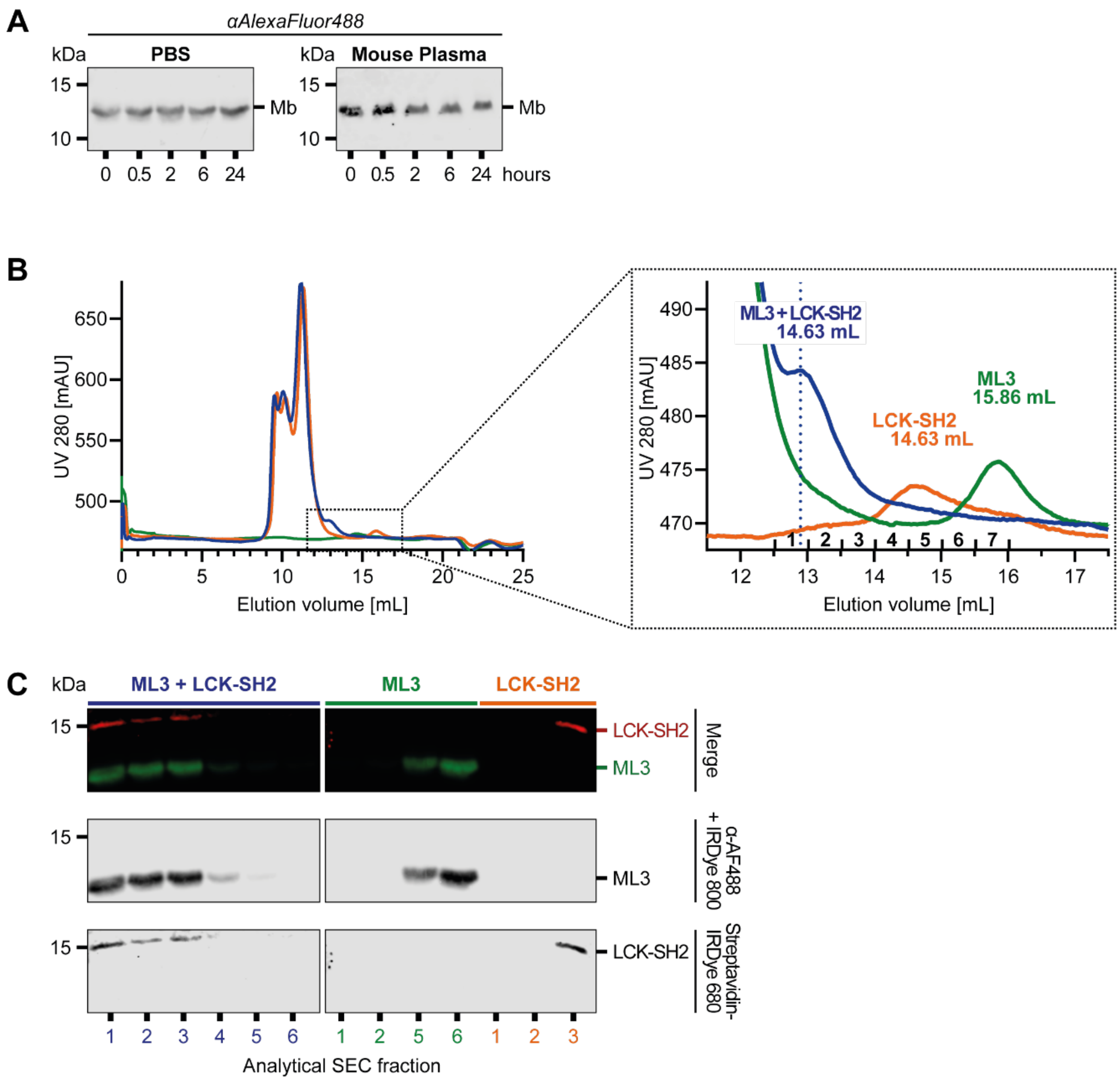

Supplementary Figure 1. Plasma Stability and Target Binding of Monobody ML3

(A) Plasma stability analysis of TEV-cleaved ML3. Purification tags were removed from ML3 using TEV protease cleavage and the monobody was C-terminally labeled with AlexaFluor 488 before incubation in either PBS or mouse plasma. Samples were taken at indicated timepoints and analyzed by SDS-PAGE and immunoblotting. Levels of monobody were assessed by an anti-AlexaFluor 488 antibody. Representative immunoblots from three to six repeats are shown. (B) Analytical size exclusion chromatography analysis of complex formation of ML3 with its target, LCK-SH2, after incubation in mouse plasma. ML3 was either mixed with equimolar concentrations of LCK-SH2 (blue) or PBS (green) and eluted from a Superdex 75 Increase 10/300 GL column. LCK-SH2 alone was eluted without prior incubation in plasma (orange). The dotted rectangle in the left chromatogram indicates area shown as close-up on the right. *mAU*: milli-absorbance units. (C) Immunoblot of fractions sampled from analytical SEC from (B). AlexaFluor 488-labeled ML3 and biotinylated LCK-SH2 domain were detected by an anti-AlexaFluor 488 antibody + IRDye800 secondary antibody (middle panel) and Streptavidine-IRDye680 (lower panel). Merge shows both channels combined (upper panel, red = LCK-SH2, green = ML3).

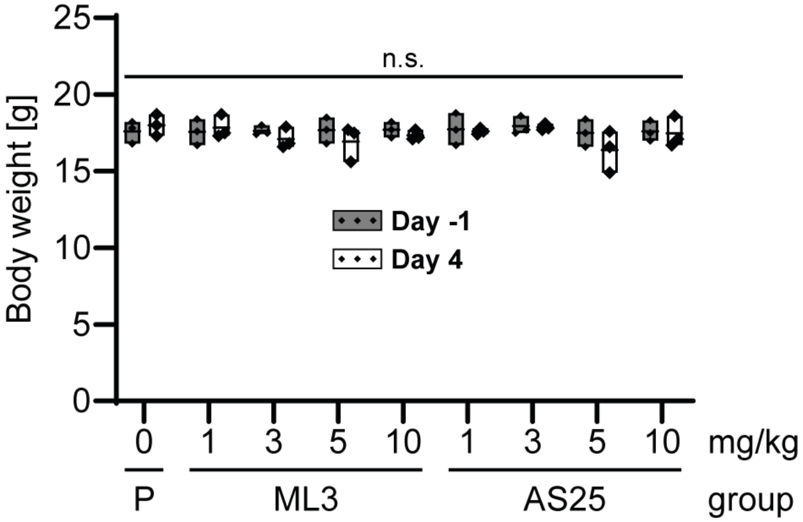

Supplementary Figure 2. Body weights of BALB/c mice injected with Monobodies

Body weight measurements of BALB/c mice after single dose-injection of vehicle control (PBS, P), monobody ML3 or monobody AS25 at 1, 3, 5, 10 mg/kg, respectively. Mice were weighed one day prior to injection (Day -1) and at the end of the period observed in this study (Day 4). Body weight is plotted as *Mean* (line) with *Min-to-max (*boxes*)* and *individual body weights (*dots*)*, *n = 3*. Group means and standard deviations (*SD*) were calculated and statistical tests conducted as 2-sided tests with level of significance set to *0.05* or *P≤0.05*.

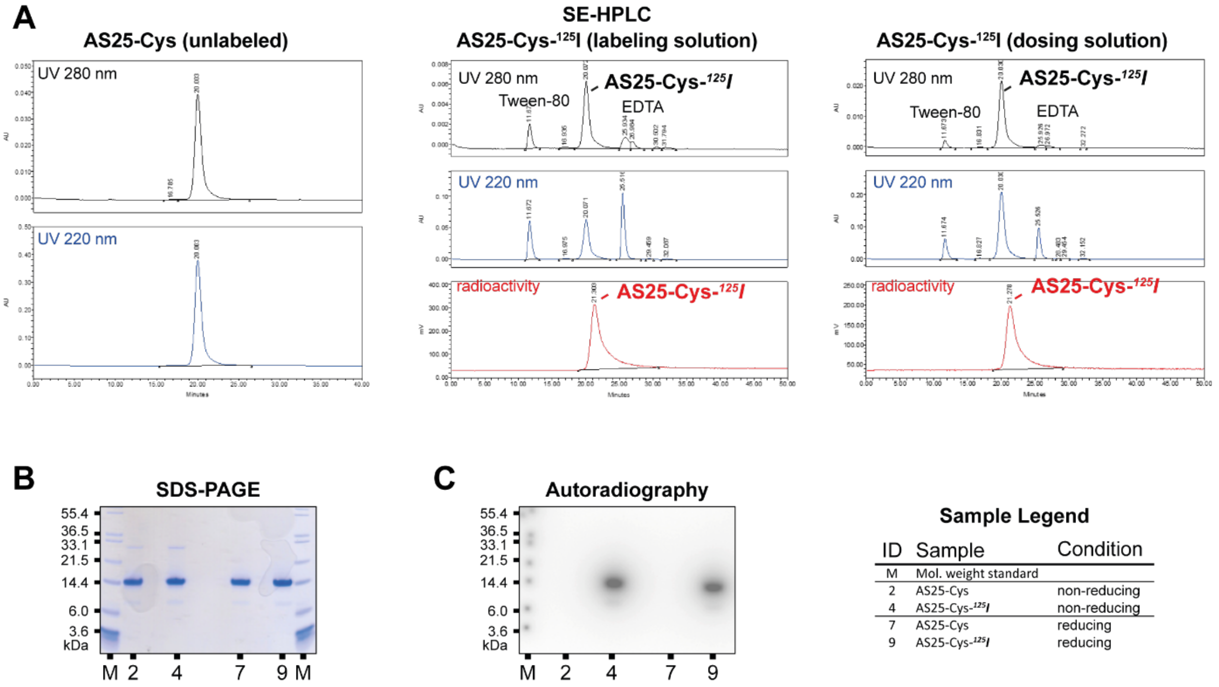

Supplementary Figure 3. Quality control of unlabeled and Iodine-125 labeled AS25

(A) SE-HPLC chromatograms of AS25 before labeling (left panel), after labeling (mid panel) and dosing solution prepared by isotopic dilution (right panel). Respective solutions were eluted from a Superdex increase 75 10/300 GL column using a Waters alliance HPLC system equipped with a spectrometer set to 280 nm and 220 nm and a Radiomatric C150TR flow scintillation analyzer. (B)-(C) SDS-PAGE (B) and Autoradiography (C) assessment of AS25 before and after labeling. Proteins were mixed with reducing or non-reducing sample buffer and heated at 65°C for 5 minutes, followed by separation using NuPAGE Novex 12% Bis-Tris Gels (NP0321BOX, Life technologies). Total protein was stained using Coomassie Blue and radioactivity detected by exposition to a phosphorus screen developed in a Typhoon IP (Amersham).

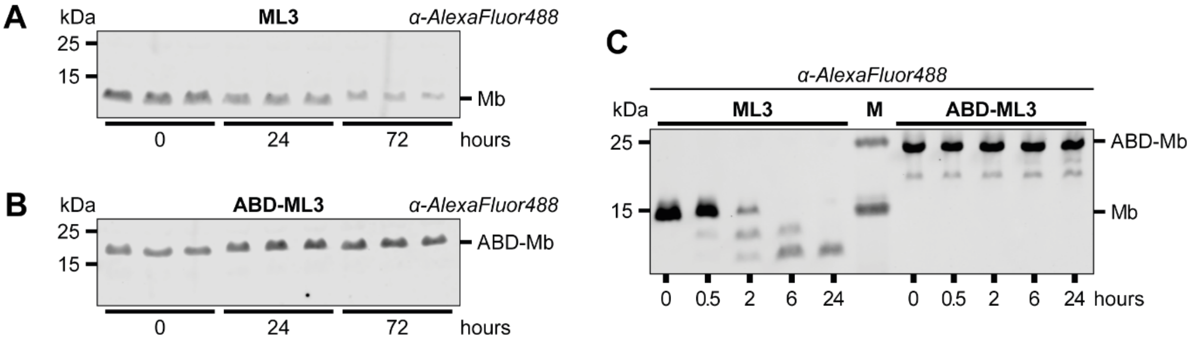

Supplementary Figure 4. Plasma Stability comparison of ML3 and ABD-ML3

(A)-(B) Representative immunoblots of the plasma stability of TEV-cleaved ML3 (A, Mb) and ABD-ML3 (B, ABD-Mb). Purification tags were removed from ML3 and ABD-ML3 using TEV protease cleavage and the monobodies were C-terminally labeled with AlexaFluor 488 before incubation in mouse plasma. Samples were taken at the indicated timepoints and analyzed by SDS-PAGE and immunoblotting. Levels of monobody were determined by an anti-AlexaFluor 488 antibody.
(C) Representative immunoblot of plasma stability comparison of uncleaved ML3 (Mb) and ABD-ML3 (ABD-Mb). The monobodies were incubated in mouse plasma at 37°C. Samples were taken at indicated timepoints and analyzed by SDS-PAGE and immunoblotting. Levels of monobody were determined by an anti-AlexaFluor 488 antibody.

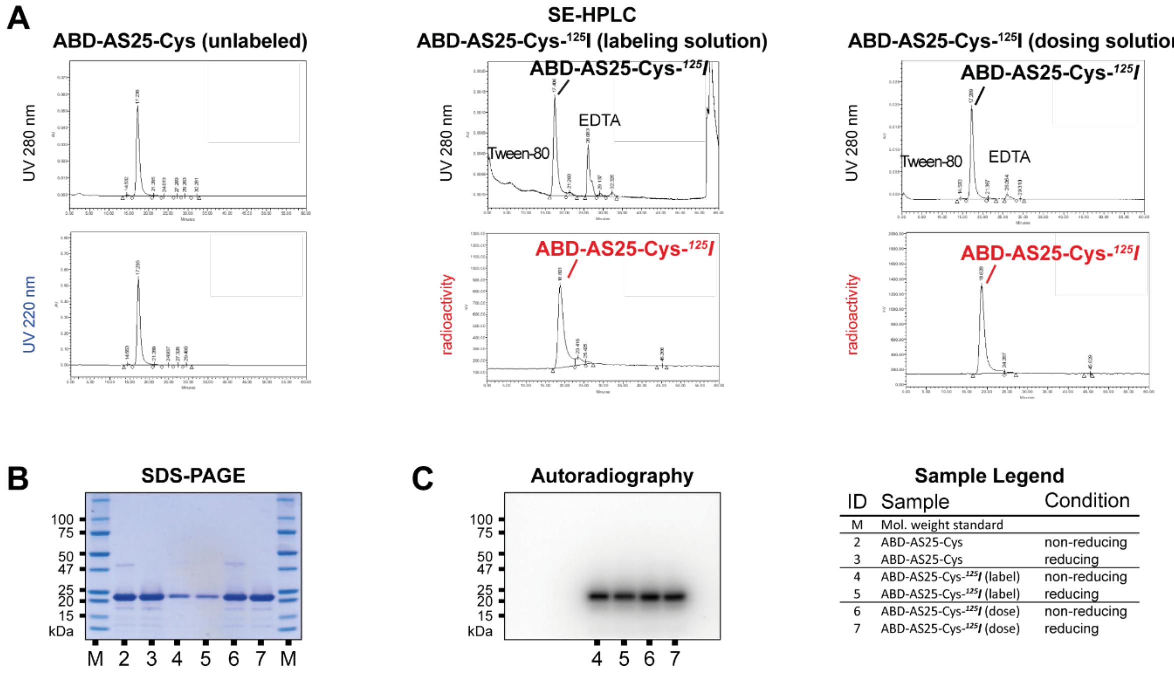

Supplementary Figure 5. Quality control of unlabeled and Iodine-125 labeled ABD-AS25

(A) SE-HPLC chromatograms of ABD-AS25 before labeling (left panel), after labeling (mid panel) and dosing solution prepared by isotopic dilution (right panel). Respective solutions were eluted from a Superdex increase 75 10/300 GL column using a Waters alliance HPLC system equipped with a spectrometer set to 280 nm / 220 nm and a radio detector Berthold LB 513. (B)-(C) SDS-PAGE (left panel) and Autoradiography (mid panel) assessment of ABD-AS25 before and after labeling. Proteins were mixed with reducing or non-reducing sample buffer and heated at 95°C for 5 minutes, followed by separation using Mini-PROTEAN TGX 4-15% (4561083, Biorad). Total protein was stained using Coomassie Blue and radioactivity detected by exposition to a phosphorus screen developed in a Typhoon IP (Amersham).

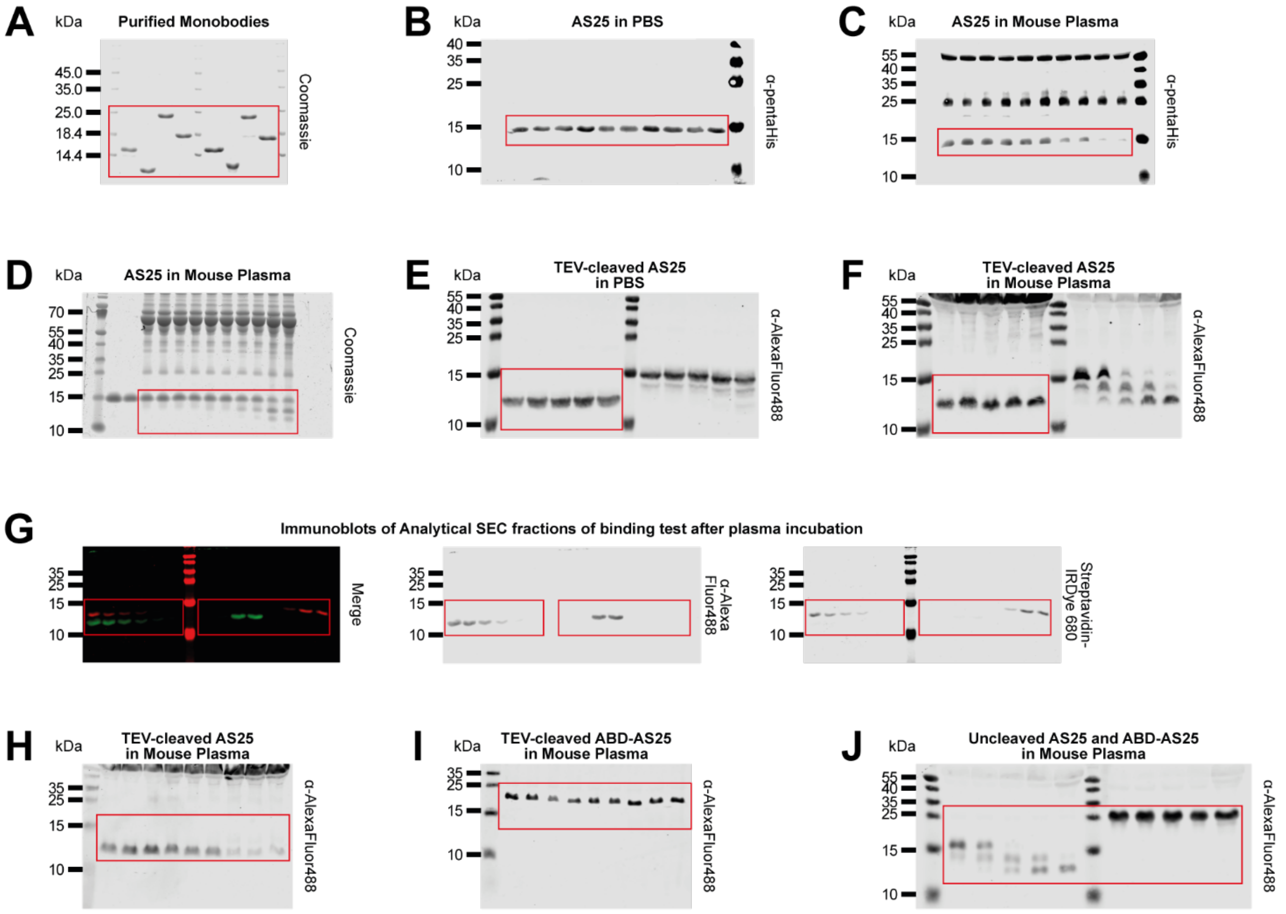

Supplementary Figure 6. Uncropped SDS-PAGE and immunoblot images used in this work

Areas used in main figures are marked with red rectangles. (A) Uncropped scan of SDS-PAGE Coomassie gel used for figure 1B. (B)-(C) Uncropped scans of anti-pentaHis + IRDye 680 immunoblots used for figures 1C and 1D. (D) Uncropped scan of SDS-PAGE Coomassie gel used for figure 1E. (E)-(F) Uncropped scans of anti-AlexaFluor 488 + IRDye 800 immunoblots used for figure 1F. (G) Uncropped scans of immunoblots used for figure 1H. *left blot*: Merge of both detection channels, *middle blot*: anti-AlexaFluor 488 + IRDye 800 immunoblot detected in 800 nm channel, *right blot*: Streptavidin-IRDye 680 immunoblot detected in 700 nm channel. (H) Uncropped scan of anti-AlexaFluor 488 + IRDye 800 immunoblot used for image 3A. (I) Uncropped scan of anti-AlexaFluor 488 + IRDye 800 immunoblot used for image 3B. (J) Uncropped scan of anti-AlexaFluor 488 + IRDye 800 immunoblot used for image 3C.

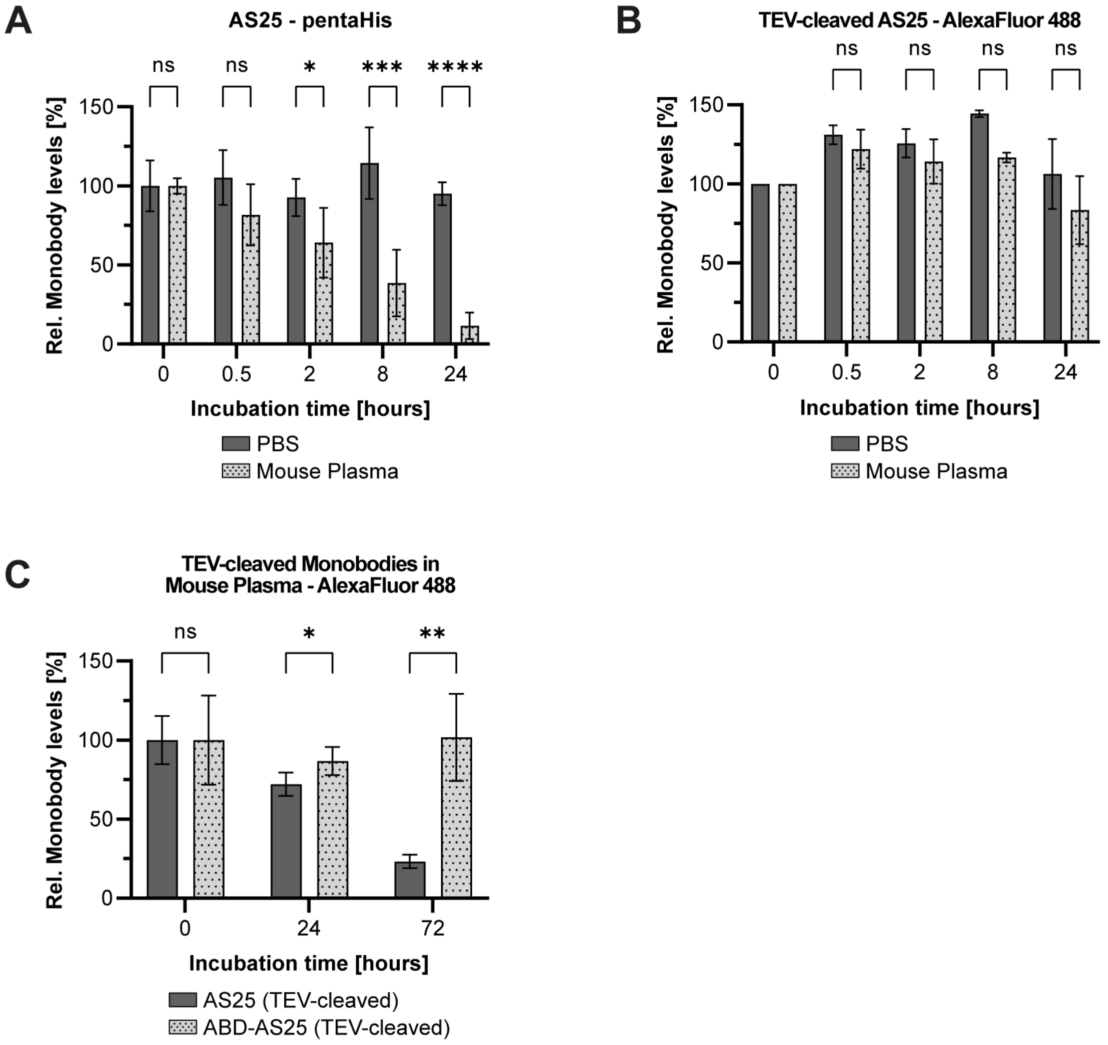

Supplementary Figure 7. Statistical analysis of plasma stability of monobodies

(A) Relative levels of AS25 after incubation in PBS or mouse plasma as detected by pentaHis immunoblotting. Relative monobody levels were calculated across three to five blots in relation to t0 samples (0 hours) of the respective condition, which were set to 100% percent, and are plotted as *Mean ± SD*. Statistical tests were conducted as *multiple unpaired t-tests (with correction for multiple comparisons using the Holm-Šídák method)* with significance level set to *adjusted P≤0.05* using GraphPad Prims 10.0.0, *ns = not significant, ∗ = 0.0332, ∗∗ = 0.0021, ∗∗∗ = 0.0002, ∗∗∗∗ = <0.0001.* (B) Relative levels of TEV-cleaved AS25 after incubation in PBS or mouse plasma as detected by anti-AlexaFluor 488 immunoblotting. Relative monobody levels were calculated across three to five blots in relation to t0 samples (0 hours) of the respective condition, which were set to 100% percent, and are plotted as *Mean ± SD*. Statistical tests were conducted as *multiple unpaired t-tests (with correction for multiple comparisons using the Holm-Šídák method)* with significance level set to *adjusted P≤0.05* using GraphPad Prims 10.0.0, *ns = not significant, ∗ = 0.0332, ∗∗ = 0.0021, ∗∗∗ = 0.0002, ∗∗∗∗ = <0.0001.* (C) Relative levels of TEV-cleaved AS25 and TEV-cleaved ABD-AS25 as detected by anti-AlexaFluor 488 immunoblotting. Relative monobody levels were calculated from three samples per time point in relation to t0 samples (0 hours) of the respective condition,

which were set to 100% percent, and are plotted as *Mean* ± *SD*. Statistical tests were conducted as *multiple unpaired t-tests (with correction for multiple comparisons using the Holm-Šídák method)* with significance level set to *adjusted P≤0.05* using GraphPad Prims 10.0.0, *ns = not significant, ∗ = 0.0332, ∗∗ = 0.0021, ∗∗∗ = 0.0002, ∗∗∗∗ = <0.0001.*

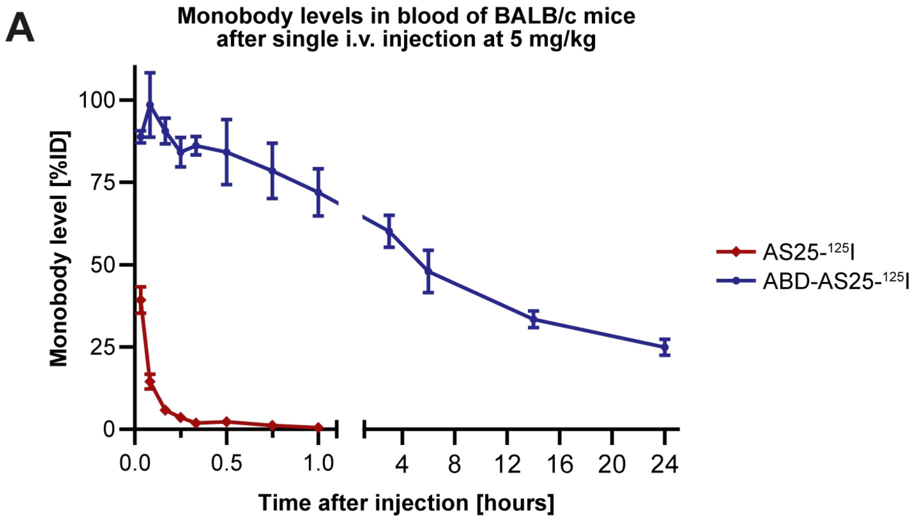

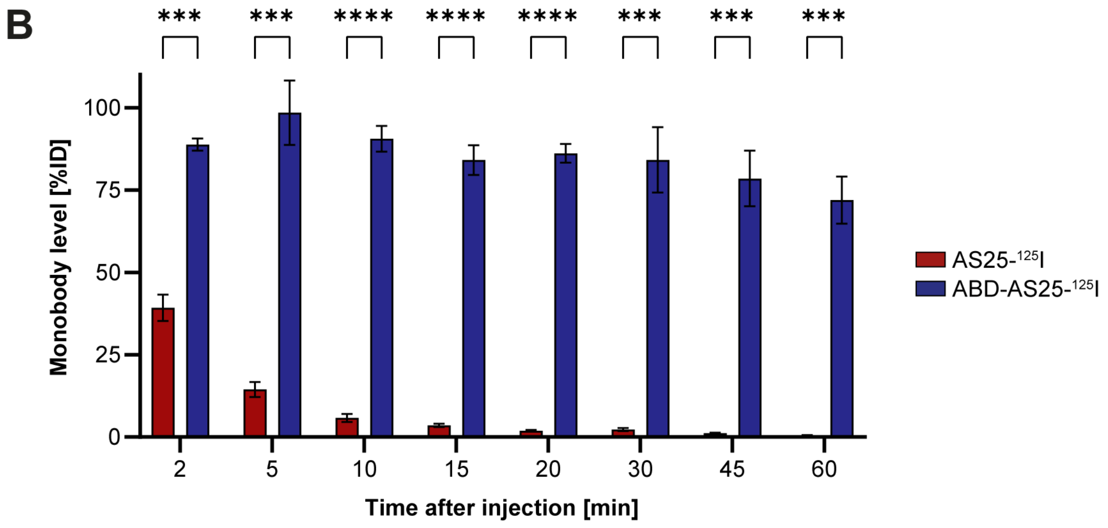

Supplementary Figure 8. Statistical analysis of blood levels of monobodies in BALB/c mice

(A) Blood levels of Iodine-125 labeled monobodies AS25 (red) or ABD-AS25 (blue) in BALB/c mice after single i.v.-injection at 5 mg/kg. Levels calculated as percentage of injected dose per total volume of blood (%ID) are taken from main figures 2A and 4A, respectively, and plotted as *Mean* ± *SD*, *n = 3.* (B) Statistical analysis of blood levels of Iodine-125 labeled monobodies within the first 60 minutes after injection. Statistical tests were conducted as *multiple unpaired t-tests (with correction for multiple comparisons using the Holm-Šídák method)* with significance level set to *adjusted P≤0.05* using GraphPad Prims 10.0.0, *ns = not significant, ∗ = 0.0332, ∗∗ = 0.0021, ∗∗∗ = 0.0002, ∗∗∗∗ = <0.0001.*

2.2 Supplementary Tables

Supplementary Table 1. Amino acid sequences of monobodies used in this work

Supplementary Table 2. Raw Data of body weight and body weight changes of BALB/c mice

Supplementary Table 3. Radiolabeling characteristics of 125I-AS25 after purification by PD-10

Supplementary Table 4. Radiolabeling characteristics of 125I-AS25 after isotopic dilution

Supplementary Table 5. Radiolabeling characteristics of 125I-ABD-AS25 after purification by PD-10

Supplementary Table 6. Radiolabeling characteristics of 125I-ABD-AS25 after isotopic dilution

Supplementary Table 1. Amino acid sequences of monobodies used in this work

Monobody name	Amino acid sequence	References
**AS25**	MKHHHHHHHHHHSSDYKDDDDKGENLYF**QG**S**SVSSVPTKLEVVAATPTSLLISWDAPAVTVDYYVITYGETGGWSGYQEFEVPGSKSTATISGLSPGVDYTITVYAYGYPYVKYNKSPISINYRTC***	(Wojcik et al., 2016)
**ABD-AS25**	MKHHHHHHHHHHSSDYKDDDDKGENLYF**QG**S**QHDEAVDANSLAEAKVLANRELDKYGVSDYYKNLINNAKTVEGVKALIDEILAALP**GGSGGGGSGGAMAS**SVSSVPTKLEVVAATPTSLLISWDAPAVTVDYYVITYGETGGWSGYQEFEVPGSKSTATISGLSPGVDYTITVYAYGYPYVKYNKSPISINYRTC***	(Stork et al., 2007; Wojcik et al., 2016)
**ML3 / Mb(Lck_3)**	MKHHHHHHHHHHSSDYKDDDDKGENLYF**QG**S**SVSSVPTKLEVVAATPTSLLISWDAPAVTVLYYLITYGETGDHWSGHQAFEVPGSKSTATISGLKPGVDYTITVYAHAESYGESYSPISINYRTC***	(Kukenshoner et al., 2017)
**ABD-ML3**	MKHHHHHHHHHHSSDYKDDDDKGENLYF**QG**S**QHDEAVDANSLAEAKVLANRELDKYGVSDYYKNLINNAKTVEGVKALIDEILAALP**GGSGGGGSGGAMA**SVSSVPTKLEVVAATPTSLLISWDAPAVTVLYYLITYGETGDHWSGHQAFEVPGSKSTATISGLKPGVDYTITVYAHAESYGESYSPISINYRTC***	(Stork et al., 2007; Kukenshoner et al., 2017)

Amino acid sequences of monobody constructs used in this work. Core monobody residues are labeled in **green**, residues of the albumin binding domain (ABD) are labeled in **violet**, Cysteine residue used for AlexaFluor 488- and Iodine-125-labeling is labeled in **orange**.

Supplementary Table 2. Raw Data of body weight and body weight changes of BALB/c mice

Group	Body weight [g]			Body weight gain
	**Animal ID**	**Day -1**	**Day 4**	**Day -1 to Day 4**
**G1 (PBS)**	2055881	18.20	18.00	-0.20
	2055882	17.80	17.30	-0.50
	2055883	16.80	18.70	1.90
	Mean	17.60	18.00	0.40
	SEM	0.72	0.70	1.31
**G2 (ML3, 1 mg/kg)**	2055884	16.70	17.30	0.60
	2055885	17.60	17.50	-0.10
	2055886	18.40	18.70	0.30
	Mean	17.57	17.83	0.27
	SEM	0.85	0.76	0.35
	%Diff	-0.20	-0.90	-33.30
**G3 (ML3, 3 mg/kg)**	2055887	17.50	16.80	-0.70
	2055888	18.00	17.90	-0.10
	2055889	17.40	16.60	-0.80
	Mean	17.63	17.10	-0.53
	SEM	0.32	0.70	0.38
	%Diff	0.20	-5.00	-233.30

**G4 (ML3, 5 mg/kg)**	2055890	16.80	15.60	-1.20
	2055891	17.70	17.50	-0.20
	2055892	18.50	17.70	-0.80
	Mean	17.67	16.93	-0.73
	SEM	0.85	1.16	0.50
	%Diff	0.40	-5.90	-283.30
**G5 (ML3, 10 mg/kg)**	2055893	17.70	17.10	-0.60
	2055894	17.20	17.20	0.00
	2055895	18.20	17.70	-0.50
	Mean	17.70	17.33	-0.37
	SEM	0.50	0.32	0.32
	%Diff	0.60	-3.70	-191.70
**G6 (AS25, 1 mg/kg)**	2055896	16.70	17.80	1.10
	2055897	18.80	17.40	-1.40
	2055898	17.70	17.60	-0.10
	Mean	17.73	17.60	-0.13
	SEM	1.05	0.20	1.25
	%Diff	0.80	-2.20	-133.30
**G7 (AS25, 3 mg/kg)**	2055899	17.50	17.70	0.20
	2055900	17.70	17.80	0.10

	2055901	18.60	18.10	-0.50
	Mean	17.93	17.87	-0.07
	SEM	0.59	0.21	0.38
	%Diff	1.90	-0.70	-116.70
**G8 (AS25, 5 mg/kg)**	2055902	17.50	16.60	-0.90
	2055903	18.40	17.60	-0.80
	2055904	16.60	14.90	-1.70
	Mean	17.50	16.37	-1.13
	SEM	0.90	1.37	0.49
	%Diff	-0.60	-9.10	-383.30
**G9 (AS25, 10 mg/kg)**	2055905	17.00	16.70	-0.30
	2055906	17.50	17.10	-0.40
	2055907	18.30	18.60	0.30
	Mean	17.60	17.47	-0.13
	SEM	0.66	1.00	0.38
	%Diff	0.00	-3.00	-133.30

Supplementary Table 3. Radiolabeling characteristics of ^{125}I-AS25 after purification by PD-10

^{125}I-AS25 (Radiolabeling solution)		
Radiolabeling yield (determined by iTLC)	%	95.0
Purification yield (after PD-10)	%	87.8
Concentration	µg/mL	495
Specific Activity	MBq/mg<br>mCi/mg	51.06<br>1.38
Moles of Iodine-125 per mol of protein		0.009
Volumic Activity	mCi/mL	0.69
Radiopurity at D0	%	99.30

Supplementary Table 4. Radiolabeling characteristics of ^{125}I-AS25 after isotopic dilution

^{125}I-AS25 (Dosing solution)		
Concentration	µg/mL	1000
Specific Activity	MBq/mg<br>mCi/mg	9.40<br>0.254
Moles of Iodine-125 per mol of protein		0.0016
Volumic Activity	mCi/mL	0.254
Radiopurity at D0	%	99.87

Supplementary Table 5. Radiolabeling characteristics of ^{125}I-ABD-AS25 after purification by PD-10

**^{125}I-ABD-AS25 (Radiolabeling solution)**		
**Radiolabeling yield (determined by iTLC)**	**%**	**96.97**
**Purification yield (after PD-10)**	**%**	**96.02**
**Concentration**	**µg/mL**	**344**
**Specific Activity**	**MBq/mg**<br>**mCi/mg**	**49.58**<br>**1.34**
**Moles of Iodine-125 per mol of protein**		**0.013**
**Radioactive concentration**	**mBq/mL**<br>**mCi/mL**	**17.17**<br>**0.46**
**Radiopurity at D0**	**%**	**99.91**

Supplementary Table 6. Radiolabeling characteristics of ^{125}I-ABD-AS25 after isotopic dilution

**^{125}I-ABD-AS25 (Dosing solution)**		
**Concentration**	**µg/mL**	**1000**
**Specific Activity**	**MBq/mg**<br>**mCi/mg**	**8.92**<br>**0.24**
**Moles of Iodine-125 per mol of protein**		**0.0023**
**Radioactive concentration**	**mBq/mL**<br>**mCi/mL**	**8.92**<br>**0.24**
**Radiopurity at D0**	**%**	**99.92**

3 Supplementary References

Kukenshoner, T., Schmit, N.E., Bouda, E., Sha, F., Pojer, F., Koide, A., et al. (2017). Selective Targeting of SH2 Domain-Phosphotyrosine Interactions of Src Family Tyrosine Kinases with Monobodies. *J Mol Biol* 429(9)**,** 1364-1380. doi: 10.1016/j.jmb.2017.03.023.

Stork, R., Muller, D., and Kontermann, R.E. (2007). A novel tri-functional antibody fusion protein with improved pharmacokinetic properties generated by fusing a bispecific single-chain diabody with an albumin-binding domain from streptococcal protein G. *Protein Eng Des Sel* 20(11)**,** 569-576. doi: 10.1093/protein/gzm061.

Wojcik, J., Lamontanara, A.J., Grabe, G., Koide, A., Akin, L., Gerig, B., et al. (2016). Allosteric Inhibition of Bcr-Abl Kinase by High Affinity Monobody Inhibitors Directed to the Src Homology 2 (SH2)-Kinase Interface. *J Biol Chem* 291(16)**,** 8836-8847. doi: 10.1074/jbc.M115.707901.
